# Adjunctive Medical Therapy with α-Blocker after Extracorporeal Shock Wave Lithotripsy of Renal and Ureteral Stones: A Meta-Analysis

**DOI:** 10.1371/journal.pone.0122497

**Published:** 2015-04-10

**Authors:** Mingchao Li, Zhengyun Wang, Jun Yang, Xiaolin Guo, Tao Wang, Shaogang Wang, Chunping Yin, Jihong Liu, Zhangqun Ye

**Affiliations:** 1 Department of Urology, Tongji Hospital, Tongji Medical College, Huazhong University of Science and Technology, Wuhan, 430030, Hubei, P. R. China; 2 Department of Respiratory and Critical Care Medicine, Tongji Hospital, Tongji Medical College, Huazhong University of Science and Technology, Wuhan, Hubei, P. R. China; 3 Department of Pharmacy, Tongji Medical College, Huazhong University of Science and Technology, Wuhan, China; Sun Yat-sen University, CHINA

## Abstract

**Background:**

Although some trials assessed the efficacy and safety of the α-blocker in facilitating renal and ureteral stones expulsion after extracorporeal shock wave lithotripsy (ESWL), the role of the α-blocker in facilitating upper urinary calculi expulsion after ESWL remain controversial.

**Aims:**

To determine the efficacy and safety of the α-blocker in facilitating renal and ureteral stones expulsion after ESWL.

**Methods:**

A literature search was carried out using the PubMed database, EMBASE and the Cochrane Library database to identify relevant studies. Two reviewers independently extracted data and assessed methodological quality. Pooled effect estimates were obtained using a fixed- and random-effects meta-analysis.

**Results:**

The meta-analysis included 23 RCTs, α-blocker significantly enhanced expulsion rate of upper urinary tract calculi after ESWL (P<0.00001; RR 1.21; 95% CI 1.12–1.31), significantly promoted steinstrasse expulsion (P=0.03; RR 1.25; 95% CI 1.03–1.53), significantly shortened the discharge time of upper urinary tract calculi (P=0.0001; MD -2.12; 95% CI -3.20–-1.04), significantly reduced the patient's pain VAS score (P=0.001; RR -1.0; 95% CI -1.61–-0.39). Compared with the control group, dizziness (P=0.002; RR 5.48; 95% CI 1.91–15.77), anejaculation (P=0.02; RR 12.17; 95% CI 1.61–91.99) and headache (P=0.04; RR 4.03; 95% CI 1.04–15.72) in the α-blocker group was associated with a higher incidence.

**Conclusions:**

Treatment with α-blocker after ESWL appears to be effective in enhancing expulsion rate of upper urinary tract calculi, shortening the discharge time of upper urinary tract calculi, reducing the patient's pain. The side effects of α-blocker were light and few.

## Introduction

Urolithiasis has plagued human beings for thousands of years [1]. Urolithiasis is a disease that affects 8–15% of the population of Europe and North America [2]. Extracorporeal shock wave lithotripsy (ESWL) was introduced by Chaussy et al in the 1980s [3]. Today, about 80% of urinary tract stones are managed with ESWL. Initially a treatment for renal and upper ureteric stones, it soon became clear that ESWL could also be used to treat stones within the middle and distal ureter [4]. ESWL produces fragmentation of the calculi using shockwaves and facilitates calculi elimination through the excretory pathway, is currently the initial treatment of choice for uncomplicated stones located in the upper urinary tract [5]. Success rates of ESWL depend on the type of lithotripter used, stones size and location [6]. In recent years, new treatments have been developed aiming to further improve the success rate after ESWL. Medical expulsion therapy, which includes α-blocker, and conventional analgesic and anti-inflammatory drugs, has shown promise in accelerating the spontaneous clearance of urinary stones as well as adjunctive treatment after ESWL for urinary stone [7].

More recent studies evaluated effect of α-blocker after ESWL on urinary stones clearance, but the evidence for their effectiveness in assisting stones clearance remained conflicting. A meta-analysis combining the studies reported to date would provide information about effect of α-blocker. The direction and magnitude of this effect will help in guiding decisions about clinical practice.

## Methods

### Search strategy

The literature search was undertaken according to the guidelines of the Centre for Reviews and Dissemination and Preferred Reporting Items for Systematic Reviews and Meta-analysis (PRISMA) statement [8]. An extensive PubMed, EMBASE, and The Cochrane Library search was performed including the following terms: α-blocker (or α-adrenergic antagonist, or α receptor antagonist, or tamsulosin, or doxazosin, or alfuzosin, or terazosin), and SWL (or ESWL, or shock wave lithotripsy, or shockwave lithotripsy, or ultrasonic lithotripsy, or lithotripter). We considered all publications in any language published before February 28, 2014.

### Study selection

The studies that met the following criteria were included: (1) RCTs; (2) patients with renal and/or ureteric calculi who were treated with ESWL; (3) α-blocker as an intervention compared with placebo or a control group; (4) Outcome measures that should be reported were clearance rate or pain (VAS) or expulsion time. Exclusion criteria were: trials in which combined intervention of α-blocker with other proven spasmolytics (e.g. corticosteroids, calcium channel blockers and phloroglucinol) were applied.

### Data abstraction and quality assessment

The abstraction of data was conducted by two independent investigators. Discrepancies were resolved by discussion and simultaneous reference to the relevant literatures. The methodological quality of the included trials was evaluated using the Jadad quality scale [9]: (1) randomization (the study was described as randomized); (2) double blinding (participant masking and researcher masking); (3) reporting of the number of dropouts and reasons for withdrawal; (4) allocation concealment; (5) generation of random numbers (by using computer, random numbers table, shuffled cards, or tossed coins). RCTs scored 1 point for each area addressed in the study design for a possible score between 0 and 5 (highest level of quality). The quality of all included studies was assessed by two investigators and the articles were classified as high-quality if their Jadad score ≥4 and low quality if their Jadad score ≤3. Disagreements regarding methodological quality were resolved with discussion between reviewers.

### Statistical analysis

Meta-analyses and forest plots were carried out by the use of Review Manager version 5.3 software. RR and 95% CI were calculated for the expulsion rate of stones and incidence of side effects during treatment. Weighted mean differences and 95% CI were for expulsion time and Pain. Heterogeneity was assessed using the I-square test. When heterogeneity was present (I-square >25%) the data was analyzed using the random-effects model, otherwise a fixed-effect was used. For all studies analyzed, a P-value of less than 0.05 was considered statistically significant. Publication bias was explored via a funnel-plot analysis. The Begg rank correlation and Egger weighted regression test methods were also used to statistically assess publication bias by Stata 12.0 (P<0.05 was consider as indicative of statistically significant publication bias). In case of heterogeneity, subgroup analysis was conducted according to dosage of drug, different stone size and location.

## Results

### Study selection and characteristics

**Fig 1:**
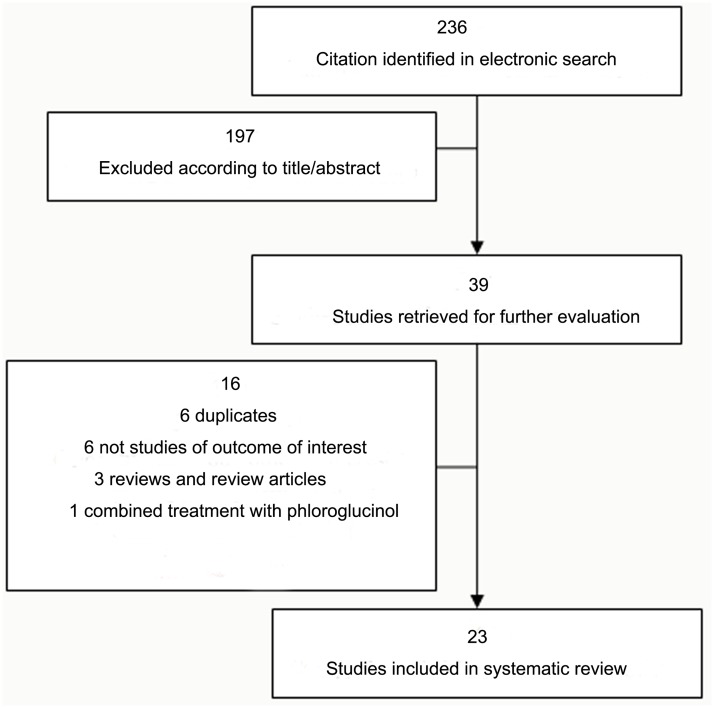
Study selection process for trials included in meta-analysis.

Our search strategy identified 236 studies, through an abstract review we excluded all references related to other topics, editorials, alternate study designs (ie observational studies), duplicate references, reviews and review articles, of which 24 were potentially relevant trials (Fig 1). One [10] was excluded for combined intervention of α-blocker with phloroglucinol (spasmolytic drug) resulting in a total of 23 RCTs [11–33] which met study criteria. The characteristics and results of the 23 included studies are summarized in Table 1.

**Table 1:** The characteristics and results of the 23 included studies.

Author (year)	Region	Subgroup	Mean age (years)	Male: female	No. patients	Stone location	Stone size range (mm)
Wang (2009)	Taiwan	TG	-	44:25	69	LU	-
		CG	51.98±8.9	25:13	38	LU	6.5±1.2
Vicentini (2011)	Brazil	TG	47.3±11.5	16:22	38	Renal	10 (5–20)
		CG	45.7±15.1	24:14	38	Renal	12 (6–20)
Georgiev (2011)	Bulgaria	TG	54±20	67:32	99	U, R	10±4,14±6
		CG	51±22	54:33	87	U, R	9±5,12±7
Falahatkar (2011)	Iran	TG	45.5±14	53:22	75	U, R	13.22
		CG	47±14	52:23	75	U, R	12.88
Agarwal (2009)	India	TG	32.4±8.7	15:5	20	UU	9.4±1.9
		CG	35.5±15.4	16:4	20	UU	10.4±3
Singh (2011)	India	TG	32.2±12.22	44:15	59	UU	-
		CG	36±13.78	41:17	58	UU	-
Resim (2005)	Turkey	TG	39(21–55)	21:11	32	LU	21(10–30)
		CG	37(23–57)	22:13	35	LU	20(10–26)
Moursy (2010)	Egypt	TG	35.6±9.95	28:16	44	U	6.39±0.99
		CG	33.9±9.71	27:17	44	U	6.07±1.18
Cakıroglu (2013)	Turkey	TG	44.66±13.25	47:12	59	U	11.40±3.01
		CG	42.19±13.17	51:13	64	U	10.70±3.2
KÜPELI (2004)	Turkey	TG	-	-	39	LU	-
		CG		-	39	LU	-
Micali(2007)	Italy	TG	45(27–71)	16:12	28	LU	10.25±1.35
		CG	46(25–72)	11:10	21	LU	9.9±1.37
Bhagat (2006)	India	TG	35.9±7.8	22:7	29	U, R	-
		CG	42.3±12.3	24:5	29	U, R	-
Kobayashi (2008)	Japan	TG	57.76±8.69	-	38	U	10.61±4.45
		CG	52.29±14.63	-	34	U	9.85±3.13
Naja (2008)	India	TG	37.17±12.59	36:15	51	R	12.12±3.59
		CG	39.44±14.49	43:22	65	R	13.06±3.49
Gravas (2007)	Greece	TG	48.8 (27–73)	18:12	30	LU	8.5 (6–13)
		CG	49.2 (30–72)	20:11	31	LU	8.3 (6–12)
Wang (2008)	China	TG	39.7±11.6	31:9	40	LU	8.6±2.6
		CG	38.5±9.5	28:12	40	LU	8.2±3.1
Ates (2012)	Turkey	TG	38.35±11.41	25:10	35	UU	9.06±1.45
		CG	30.95±9.68	33:11	44	UU	8.30±2.51
Janane (2014)	Morocco	TG	41.2 ± 12.4	108:78	186	LU	9.2 ± 2.8
		CG	43.4 ± 12.2	104:66	170	LU	9.4 ± 3.0
Hussein (2010)	Egypt	TG	44 (27–62)	40:27	67	R	-
		CG	40 (20–60)	45:24	69	R	-
Gul (2013)	Turkey	TG	63.2±6.7	-	34	U, R	12.6±5.3
		CG	58.6±7.2	-	230	U, R	13.3±4.7
Wang (2010)	China	TG	42.2±12.6	36:19	54	LU	9.3±2.6
		CG	40.9±10.3	38:14	52	LU	8.6±3.0
Cho (2012)	Korea	TG	47.4±12.6	29:12	41	U	7.1±1.7
		CG	47.7±12.1	31:12	43	U	7.2±1.8
Park (2013)	Korea	TG	46.2	29:15	44	UU	9.2
		CG	47.6	28:16	44	UU	9.6

The mean Jadad score of these 23 studies was 3.2, ranging from 1 to 5 points (Table 2). 8 of 23 RCTs met the Jadad criteria for high quality [12], [14], [16], [19]-[20], [22], [27], [29]. All of the studies included suggested randomization, and 14 studies reported the method of random sequences generation [12], [14]-[20], [22], [24], [27]-[29], [33]. Double blinded method were used only in four studies [12], [14], [16], [22], we considered that the outcomes and their measurements may likely to be influenced by lack of blinding. In general, the methodological and report qualities of the included studies were good, but still not very ideal.

**Table 2:** Jadad Trial Quality Scores.

Author (year)	Randomization	Double blinding	Withdrawal or drop-out	Total Jadad score (possible total = 5)
Wang (2009)	1	0	1	2
Vicentini (2011)	2	2	1	5
Georgiev (2011)	1	0	1	2
Falahatkar (2011)	2	2	1	5
Agarwal (2009)	2	0	1	3
Singh (2011)	2	2	1	5
Resim (2005)	2	0	1	3
Moursy (2010)	2	0	1	3
Cakıroglu (2013)	2	1	1	4
KÜPELI (2004)	2	1	1	4
Micali(2007)	1	1	1	3
Bhagat (2006)	2	2	1	5
Kobayashi (2008)	1	1	1	3
Naja (2008)	2	0	1	3
Gravas (2007)	1	1	1	3
Wang (2008)	1	0	1	2
Ates (2012)	2	1	1	4
Janane (2014)	2	0	1	3
Hussein (2010)	2	1	1	4
Gul (2013)	1	0	0	1
Wang (2010)	1	0	0	1
Cho (2012)	1	1	1	3
Park (2013)	2	0	1	3

### Statistical results

#### The expulsion rate of α-blocker for stones

**Fig 2:**
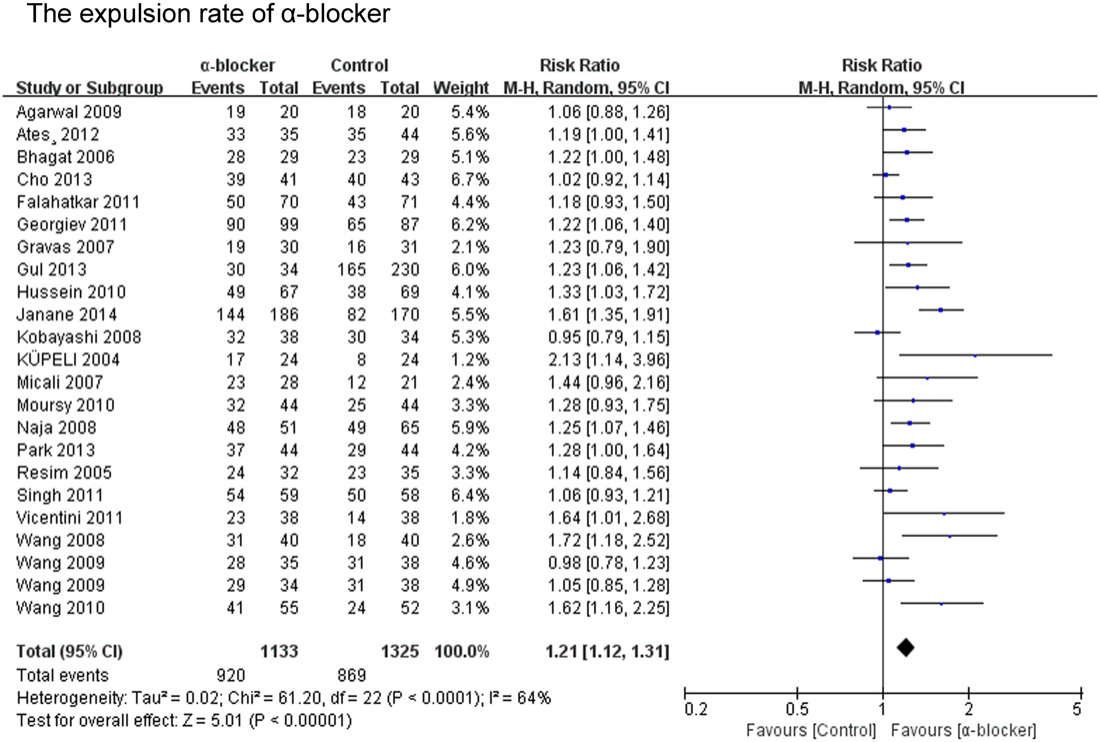
The expulsion rate of the α-blocker.

**Fig 3:**
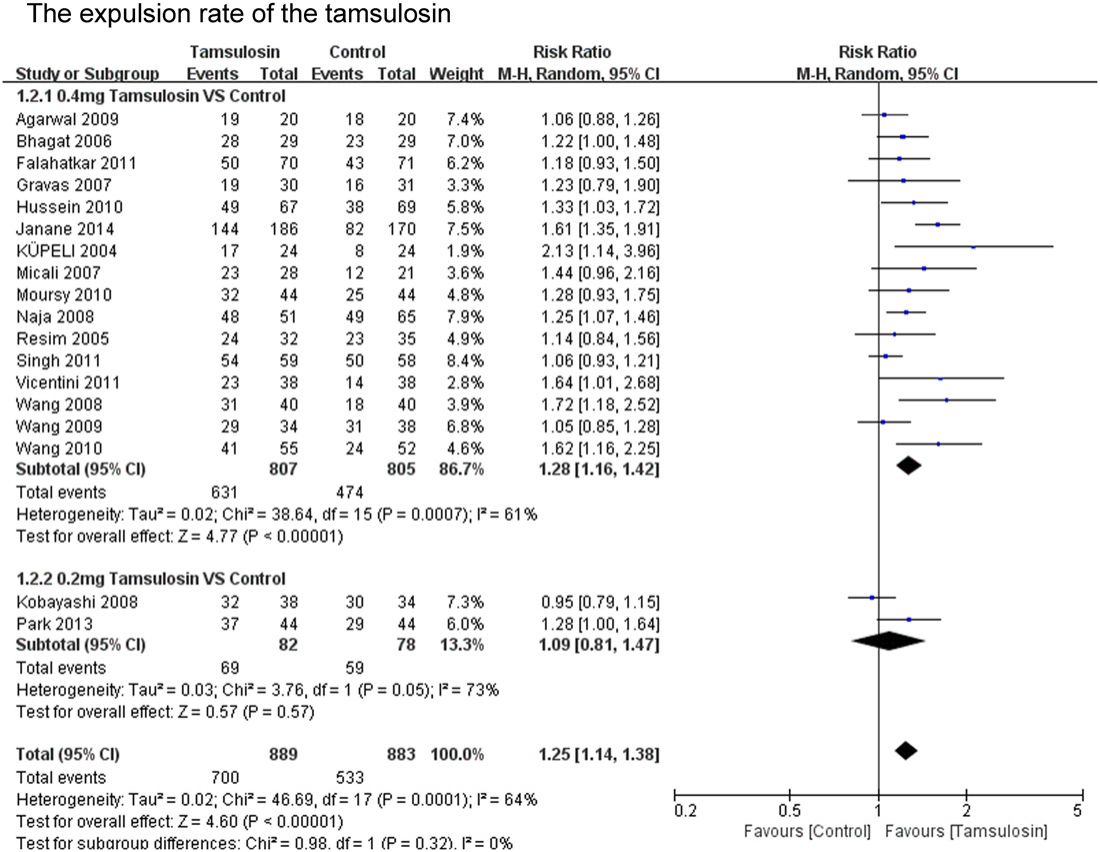
The expulsion rate of tamsulosin.

**Fig 4:**
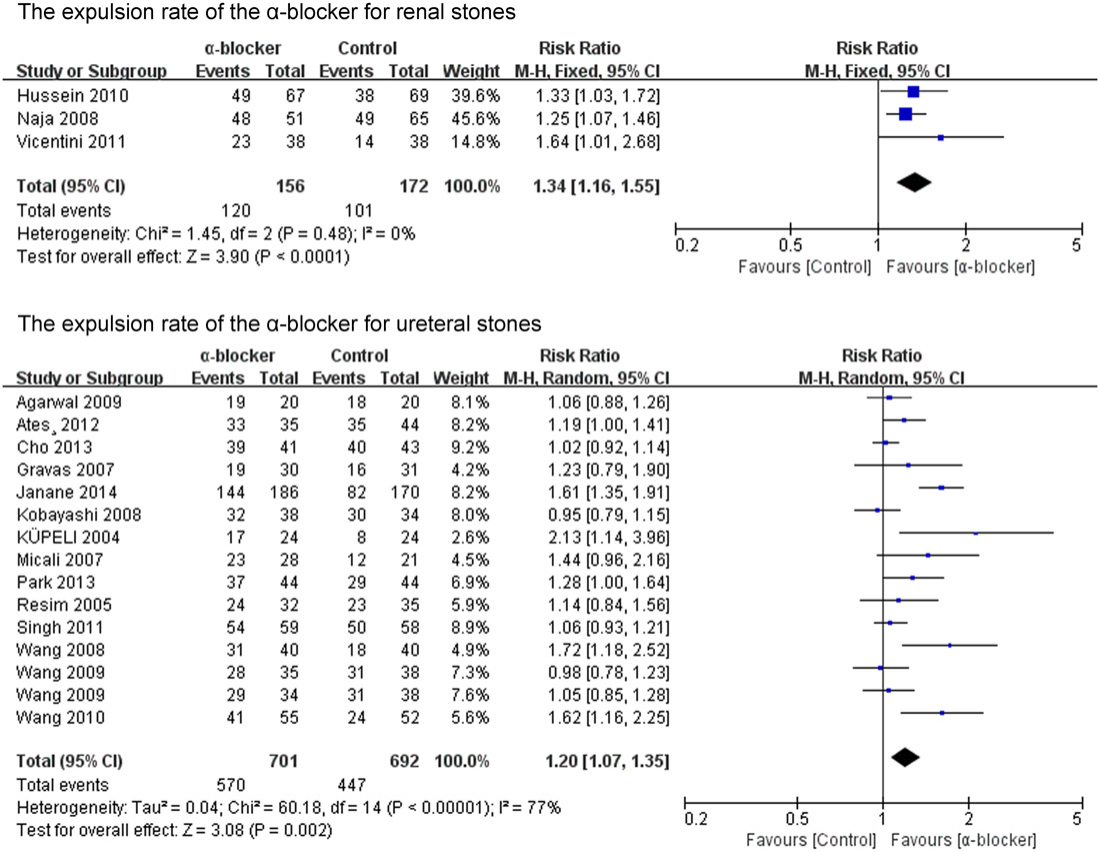
The expulsion rate of the α-blocker for renal and ureteral stones.

**Fig 5:**
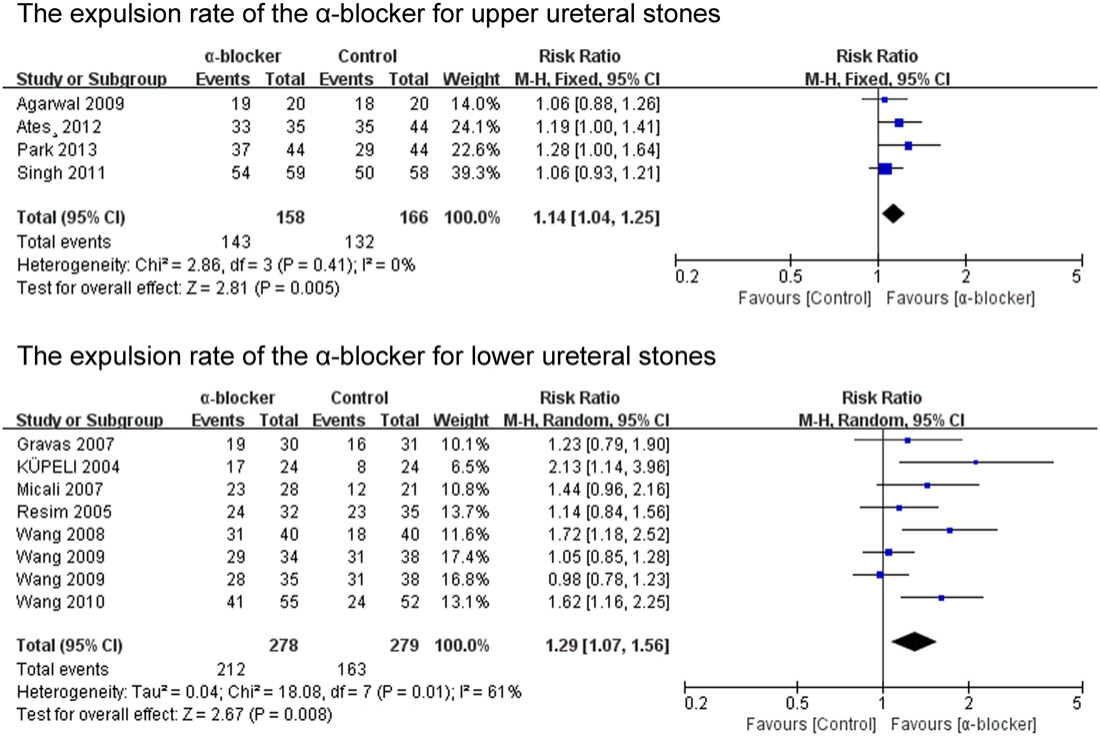
The expulsion rate of the α-blocker for upper and lower ureteral stones.

**Fig 6:**
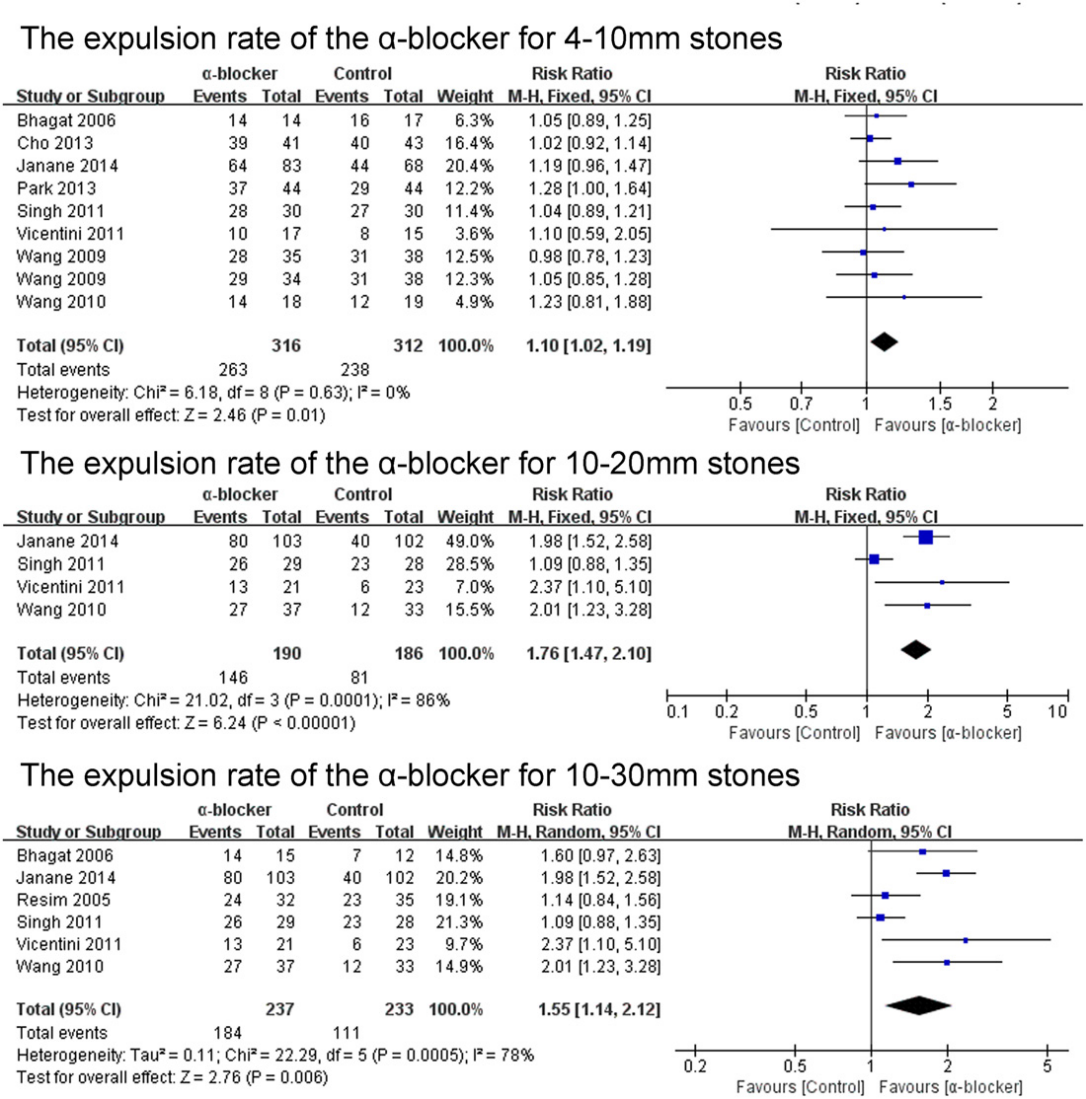
The expulsion rate of the α-blocker for different size stones.

**Fig 7:**
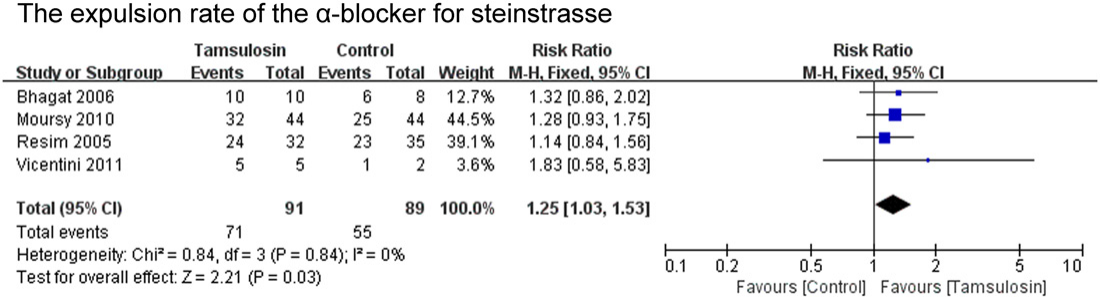
The expulsion rate of the α-blocker for steinstrasse.

The expulsion rate was analyzed in 22 of the 23 studies. The expulsion rate of the α-blocker group was significant higher than that of the control group (P<0.00001; RR 1.21; 95% CI 1.12–1.31) (Fig 2). The expulsion rate of the tamsulosin 0.4mg group was analyzed in 16 of the 23 studies. The expulsion rate of the tamsulosin 0.4mg group was significant higher than that of the control group (P<0.00001; RR 1.28; 95% CI 1.16–1.42) (Fig 3), there was no significant difference in the expulsion rate between tamsulosin 0.2mg group and control group (P = 0.57; RR 1.09; 95% CI: 0.81–1.47) (Fig 3). The expulsion rate of the α-blocker group was significant higher than that of the control group both for renal stones (P<0.0001; RR 1.34; 95% CI 1.16–1.55) and ureteral stones (P = 0.002; RR 1.20; 95% CI 1.07–1.35) (Fig 4). The expulsion rate of the α-blocker group was significant higher than that of the control group both for lower ureteral stones (P = 0.008; RR 1.29; 95% CI 1.07–1.56) and upper ureteral stones (P = 0.005; RR 1.14; 95% CI: 1.04–1.25) (Fig 5). The expulsion rate of the α-blocker group was higher than that of the control group for 4–10 mm stones (P = 0.01; RR 1.10; 95% CI: 1.02–1.19), 10–20 mm stones (P<0.00001; RR 1.76; 95% CI: 1.47–2.10) and 10–30 mm stone (P = 0.006; RR 1.55; 95% CI: 1.14–2.12) (Fig 6). The expulsion rate of the α-blocker group was significant higher than that of the control group for steinstrasse (Fig 7) (P = 0.03; RR 1.25; 95% CI: 1.03–1.53).

#### The expulsion time of the α-blocker for stones

**Fig 8:**
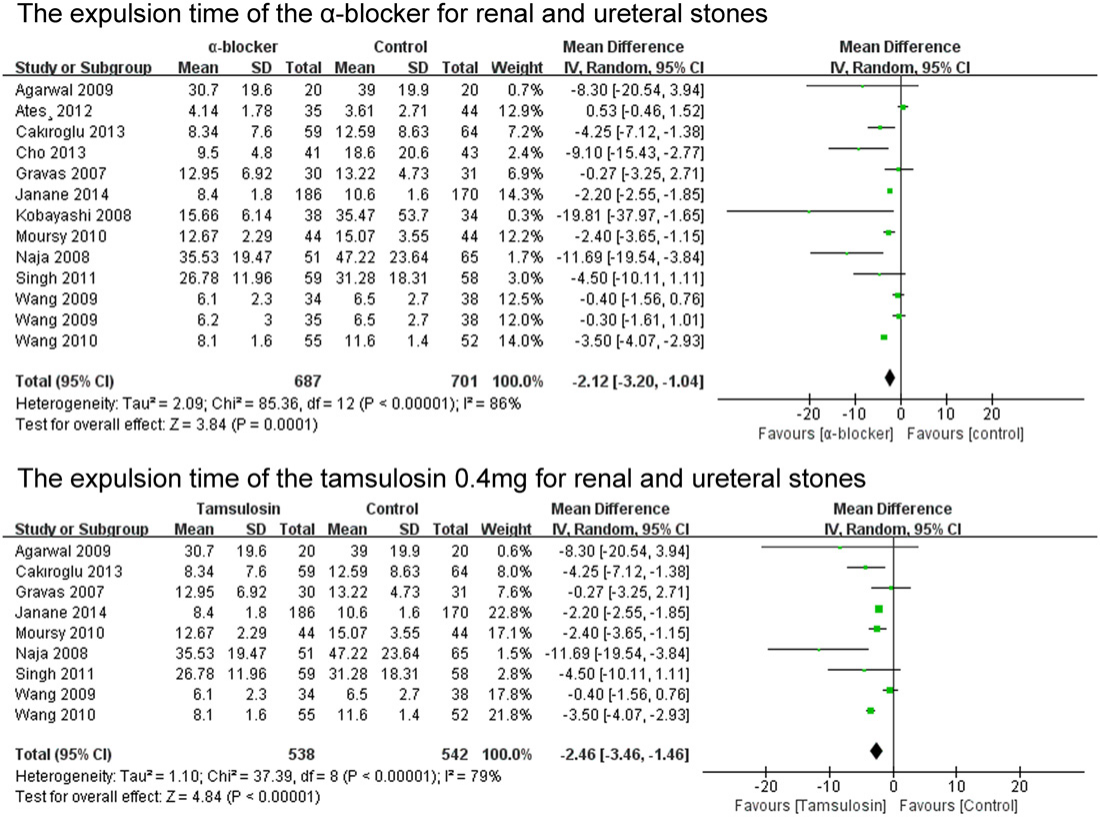
The expulsion time of the α-blocker.

**Fig 9:**
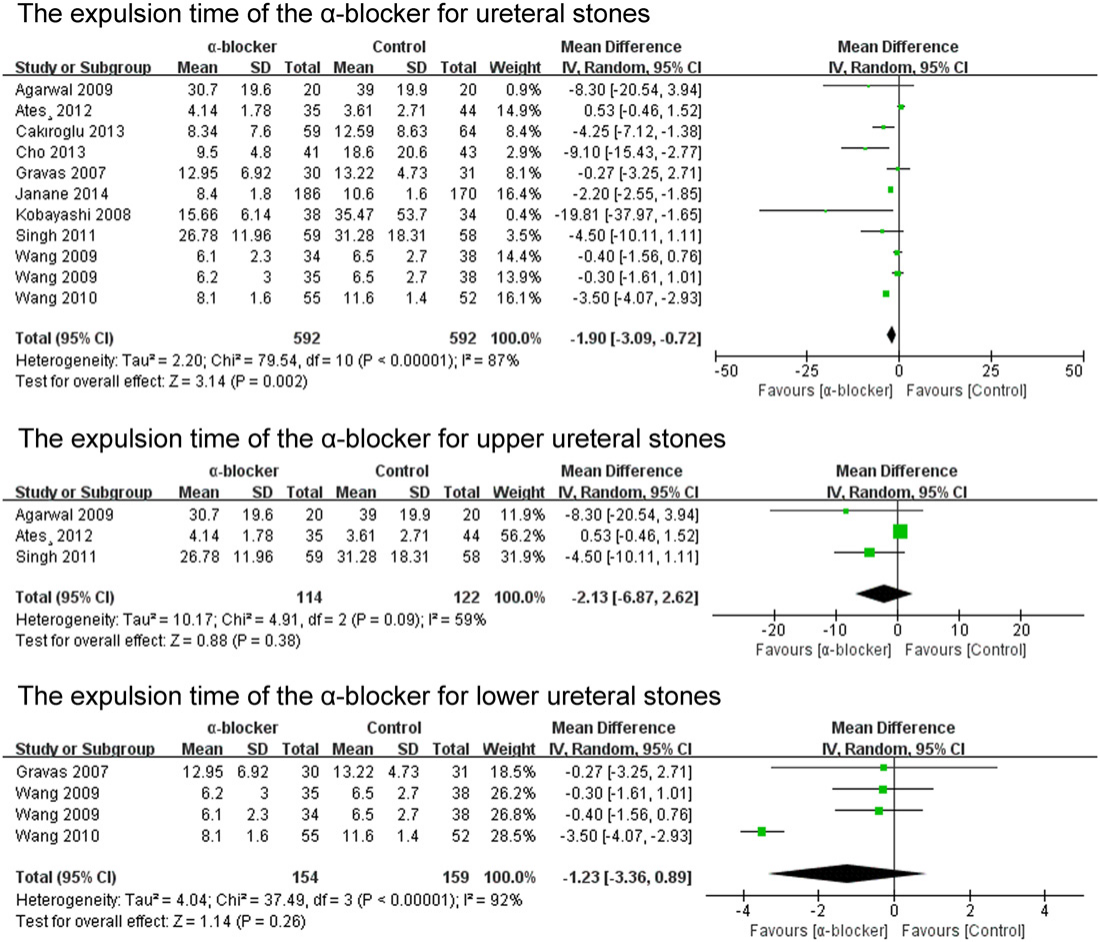
The expulsion time of the α-blocker for ureteral stones.

The expulsion time of the α-blocker group was analysed in 12 of the 23 studies. The expulsion time of the α-blocker group was significant shorter than that of the control group for renal and ureteral stones (P = 0.0001; MD -2.12; 95% CI -3.20–-1.04) (Fig 8). The expulsion time of the tamsulosin 0.4mg group was analysed in 9 of the 23 studies. The expulsion time of the tamsulosin 0.4mg group was significant shorter than that of the control group (P<0.00001; MD -2.46; 95% CI -3.46–-1.46) (Fig 8). The expulsion time of the α-blocker group was significant shorter than that of the control group for ureteral stones (P = 0.002; MD -1.90; 95% CI -3.09–-0.72) (Fig 9). There was no significant difference in the expulsion time between α-blocker group and control group both for upper ureteral stones (P = 0.38; MD -2.13 95% CI -6.87–2.62) and lower ureteral stones (P = 0.26; MD -1.23; 95% CI -3.36–0.89) (Fig 9).

#### Pain

**Fig 10:**
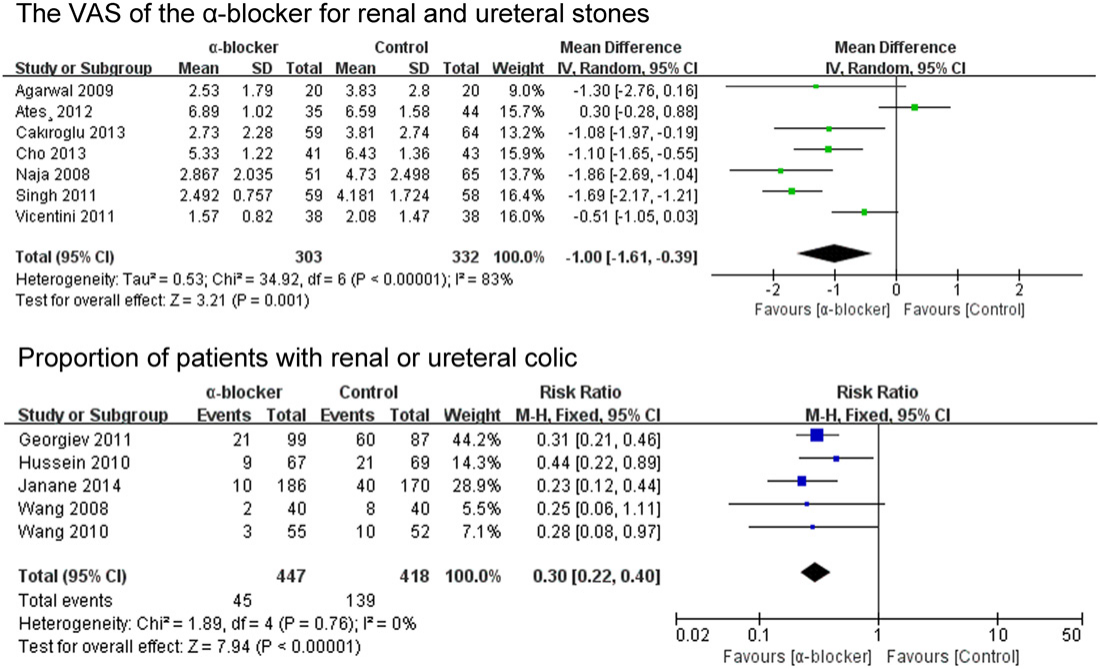
α-blocker decreasing pain.

The difference in VAS (visual analogue scale, VAS) score between the α-blocker group and control group showed statistical significance (P = 0.001; MD -1.0; 95% CI -1.61–-0.39) (Fig 10).

Proportion of patients with renal or ureteral colic of the α-blocker group was significant less than that of control group during treatment (P<0.00001; RR 0.3; 95% CI 0.22–0.40) (Fig 10).

#### Incidence of side effects during treatment

**Fig 11:**
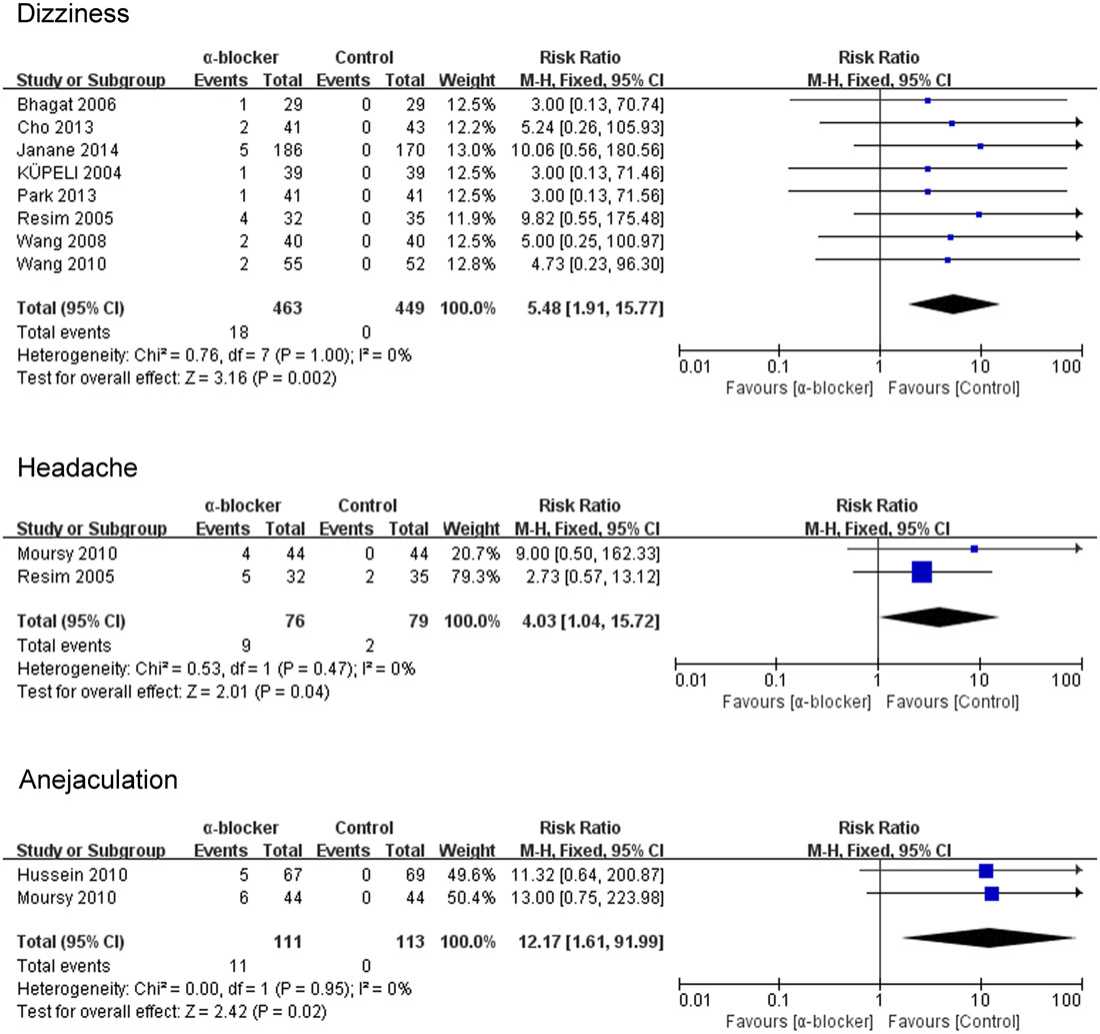
Side effects of α-blocker.

The frequencies of any adverse event are shown in Fig 11, compared with the control group, dizziness (P = 0.002; RR 5.48; 95% CI 1.91–15.77), anejaculation (P = 0.02; RR 12.17; 95% CI 1.61–91.99) and headache (P = 0.04; RR 4.03; 95% CI 1.04–15.72) in the α-blocker group was associated with a higher incidence.

### Publication bias analysis

**Fig 12:**
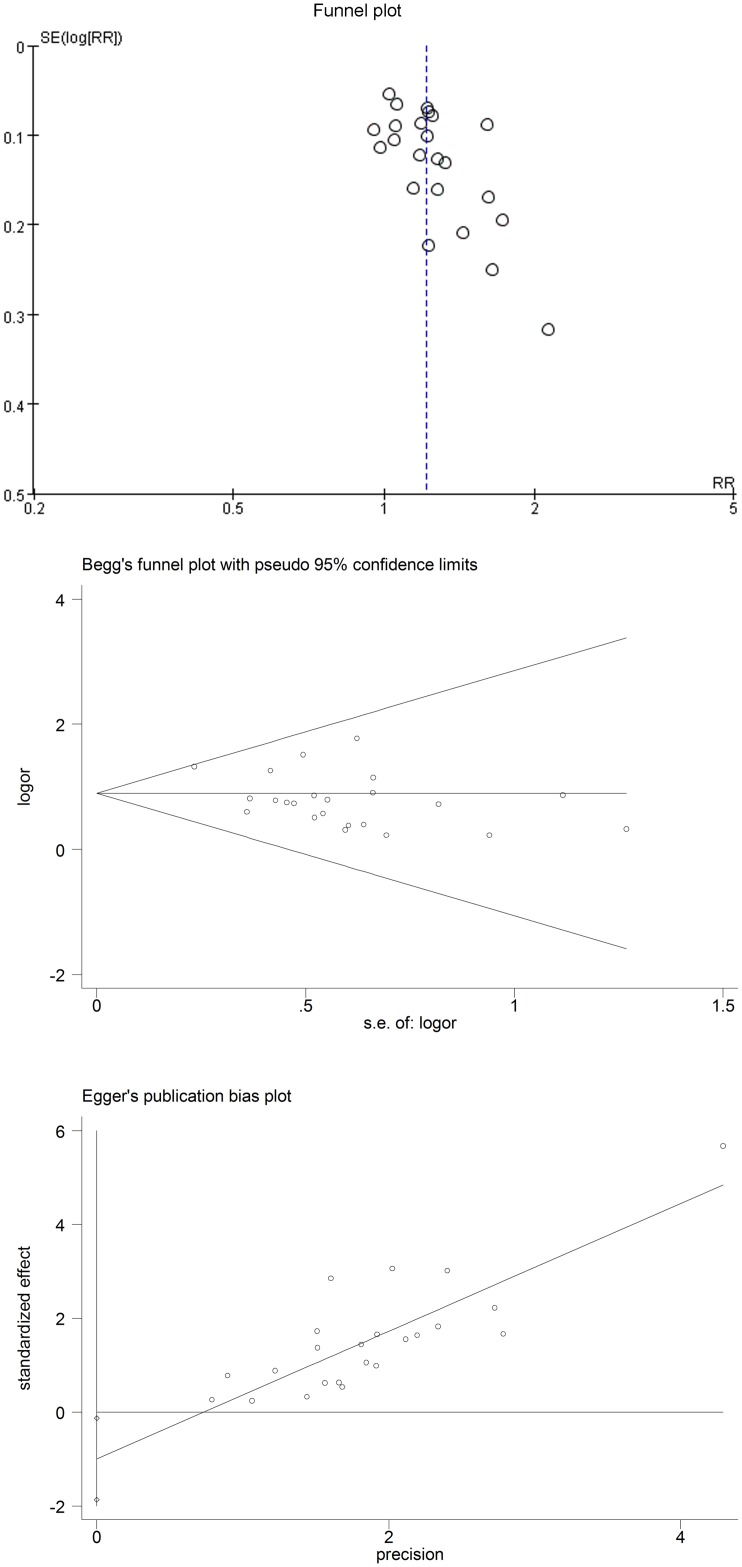
Funnel plot analysis to detect publication bias.

The distribution of the studies using traditional funnel plot (Fig 12) showed asymmetrical distribution of effect estimate, which suggested the possibility of publication bias. Egger weighted regression analysis (p = 0.027) also showed presence of publication bias. But the Begg rank correlation statistic (p = 0.428) showed no evidence of publication bias.

## Discussion

This meta-analysis suggested that α-blocker significantly enchanced the expulsion rate of upper urinary tract calculi and steinstrasse, shortened stones expulsion time, reduced the pain of patients. Side effects of α- blocker was light and few.

This meta-analysis included 23 RCTs [11]-[33], with 979 patients in experimental group, 933 patients in control group. Heterogeneity existed in expulsion rate of upper urinary tract calculi, the reason for heterogeneity might relate to ESWL energy and frequency, the location and the size of the stones. When separately analyzed ureter calculi, renal stones, 4–10 mm stones, we did not observe heterogeneity.

We observed a significant improvement in the success rates for the adjuvant use of α-blockers for ureteral stones, upper and lower ureteral stones, renal stones, 4–10 mm stones, 10–20 mm stones, 10–30mm stones. α-blocker could significantly promote steinstrasse discharge. A previous meta-analysis [6] excluded steinstrasse, our meta-analysis for the first time showed that α-blockers could significantly promoted steinstrasse discharge.

Our meta-analysis from studies suggested that α-blockers could significantly shorten the discharge time of upper urinary tract stones and ureteral stones, but there was heterogeneity among these studies. The reason for heterogeneity might relate to difference of drug treatment time, ESWL energy and frequency, the location and the size of the stones. α-blocker shorten upper and lower ureteral stones discharge time, but without statistical significance, which might be related to the size and location of the stones.

Tamsulosin was used as adjuvant therapy in 20 studies, of which 18 was tamsulosin 0.4mg [11]-[12], [14]-[18], [20]-[22], [24]-[26], [28]-[29], [31], of which 2 was tamsulosin 0.2mg, one of which was from Japan [23], another of which was from South Korea [33]. Tamsulosin 0.4 mg significantly promoted the discharge of upper urinary tract stones, significantly shorten the discharge time of stones. However, tamsulosin 0.2 mg could not significantly promote the ureteral stones expulsion, however, the result only from two studies, this need a large number of clinical trials to confirm.

α-blockers can significantly reduce the patient's pain and the proportion of patients with renal colic. In some studies, a total VAS score was 100points, in some studies, a total VAS score was 10 points, in order to facilitate statistical analysis, we took 100 points as the total score of data conversion into total score of 10 points data.

Side effect of α-blockers was light and few, the common side effects were headache, dizziness, majority of which was mild dizziness, anejaculation. In the treatment group, one patient experienced delay ejaculation, one patient experienced abnormal ejaculation, one patient experienced retrograde ejaculation, one patient experienced postural hypotension, two patients experienced diarrhea, four patients experienced rhinitis. With regarding to nausea with or without vomiting, four patients experienced in the treatment group, five patients experienced in control group.

The characteristic of this meta-analysis was statistically analyzed the effect of α-blockers on steinstrasse and on different location and size stones.

The shortcoming of this meta-analysis was that there were heterogeneities among studies. The heterogeneities might relate to different duration of treatment, different stone size and location, different ESWL energy and frequency among studies. Most of the included trials failed to describe detail information about randomization and allocation concealment. Lack of blinding procedures in RCTs can also exaggerate the conclusions of these trials. In addition, publication bias should also not be ignored because both the funnel plot and Egger’s test showed the possibility of publication bias, even though the Begg’s test showed no evidence of publication bias. Further assessment of α-blockers needs to be taken by large-scale clinical studies which employ rigorous methodologies. So the results need to be interpreted cautiously. But on the whole, to some extent, the results of this meta-analysis will help clinicians to make some right clinical decisions. As more and more clinical trials take, conclusions will be more credible.

## Supporting Information

S1 PRISMA Checklist PRISMA checklist. (DOC)

## References

[1] Cui Y, Cao W, Shen H, Xie J, Adams TS, Zhang Y, et al Comparison of ESWL and Ureteroscopic Holmium Laser lithotripsy in Management of Ureteral Stones. PLoS One.2014; 9:e87634 10.1371/journal.pone.0087634 24498344 PMC3912003

[2] Sayed MA, Abolyosr A, Abdalla MA, El-Azab AS. Efficacy of tamsulosin in medical expulsive therapy for distal ureteral calculi. Scand J Urol Nephrol. 2008; 42: 59–62. 17853008 10.1080/00365590701571076

[3] Chaussy C, Brendel W, Schmiedt E. Extracorporeally induced destruction of kidney stones by shock waves. Lancet. 1980; 2: 1265–1268. 6108446 10.1016/s0140-6736(80)92335-1

[4] Phipps S, Stephenson C, Tolley D. Extracorporeal shockwave lithotripsy to distal ureteric stones: the transgluteal approach significantly increases stone-free rates. BJU Int. 2013; 112: E129–133. 10.1111/j.1464-410X.2012.11738.x 23360696

[5] Carrasco J, Anglada FJ, Campos JP, Muntané J, Requena MJ, Padillo J. The protective role of coenzyme Q10 in renal injury associated with extracorporeal shockwave lithotripsy: a randomised, placebo-controlled clinical trial. BJU Int. 2014; 113: 942–50. 10.1111/bju.12485 24119199

[6] Zhu Y, Duijvesz D, Rovers MM, Lock TM. alpha-Blockers to assist stone clearance after extracorporeal shock wave lithotripsy: a meta-analysis. BJU Int. 2010; 106: 256–261. 10.1111/j.1464-410X.2009.09014.x 19889063

[7] Fan B, Yang D, Wang J, Che X, Li X, Wang L, et al Can tamsulosin facilitate expulsion of ureteral stones? A meta-analysis of randomized controlled trials. Int J Urol. 2013; 20: 818–830. 10.1111/iju.12048 23278872

[8] Moher D, Liberati A, Tetzlaff J, Altman DG. Reprint—preferred reporting items for systematic reviews and meta-analyses: the PRISMA statement. Phys Ther. 2009; 89: 873–880. 19723669

[9] Jadad AR, Moore RA, Carroll D, Jenkinson C, Reynolds DJ, Gavaghan DJ, et al Assessing the quality of reports of randomized clinical trials: is blinding necessary? Control Clin Trials. 1996; 17: 1–12. 8721797 10.1016/0197-2456(95)00134-4

[10] Zaytoun OM, Yakoubi R, Zahran AR, Fouda K, Marzouk E, Gaafar S, et al Tamsulosin and doxazosin as adjunctive therapy following shock-wave lithotripsy of renal calculi: randomized controlled trial. Urol Res. 2012; 40: 327–332. 10.1007/s00240-011-0410-x 21837534

[11] Wang CJ, Huang SW, Chang CH. Adjunctive medical therapy with an alpha-1A-specific blocker after shock wave lithotripsy of lower ureteral stones. Urol Int. 2009; 82:166–169. 10.1159/000200793 19322003

[12] Vicentini FC, Mazzucchi E, Brito AH, Chedid Neto EA, Danilovic A, Srougi M. Adjuvant tamsulosin or nifedipine after extracorporeal shock wave lithotripsy for renal stones: a double blind, randomized, placebo-controlled trial. Urology. 2011; 78: 1016–1021. 10.1016/j.urology.2011.04.062 21802124

[13] Georgiev MI, Ormanov DI, Vassilev VD, Dimitrov PD, Mladenov VD, Popov EP, et al Efficacy of tamsulosin oral controlled absorption system after extracorporeal shock wave lithotripsy to treat urolithiasis. Urology. 2011; 78: 1023–1026. 10.1016/j.urology.2011.01.073 21917304

[14] Falahatkar S, Khosropanah I, Vajary AD, Bateni ZH, Khosropanah D, Allahkhah A. Is there a role for tamsulosin after shock wave lithotripsy in the treatment of renal and ureteral calculi? J Endourol. 2011; 25: 495–498. 10.1089/end.2010.0439 21166579

[15] Agarwal MM, Naja V, Singh SK, Mavuduru R, Mete UK, Kumar S, et al Is there an adjunctive role of tamsulosin to extracorporeal shockwave lithotripsy for upper ureteric stones: results of an open label randomized nonplacebo controlled study. Urology. 2009; 74: 989–992. 10.1016/j.urology.2009.06.075 19883809

[16] Singh SK, Pawar DS, Griwan MS, Indora JM, Sharma S. Role of tamsulosin in clearance of upper ureteral calculi after extracorporeal shock wave lithotripsy: a randomized controlled trial. Urol J. 2011; 8: 14–20. 21404197

[17] Resim S, Ekerbicer HC, Ciftci A. Role of tamsulosin in treatment of patients with steinstrasse developing after extracorporeal shock wave lithotripsy. Urology. 2005; 66: 945–948. 16286100 10.1016/j.urology.2005.05.029

[18] Moursy E, Gamal WM, Abuzeid A. Tamsulosin as an expulsive therapy for steinstrasse after extracorporeal shock wave lithotripsy: a randomized controlled study. Scand J Urol Nephrol. 2010; 44: 315–319. 10.3109/00365599.2010.494616 20560802

[19] Basri Cakıroglu, Sinanoglu O, Mahmure Uraz. The effect of tamsulosin on pain and clearance according to ureteral stone location after shock wave lithotripsy. Curr Ther Res Clin Exp. 2013; 74: 33–35. 10.1016/j.curtheres.2012.12.003 24385155 PMC3862193

[20] Küpeli B, Irkilata L, Gürocak S, Tunç L, Kiraç M, Karaoğlan U, et al Does tamsulosin enhance lower ureteral stone clearance with or without shock wave lithotripsy? Urology. 2004; 64: 1111–1115. 15596181 10.1016/j.urology.2004.07.020

[21] Micali S, Grande M, Sighinolfi MC, De Stefani S, Bianchi G. Efficacy of expulsive therapy using nifedipine or tamsulosin, both associated with ketoprofene, after shock wave lithotripsy of ureteral stones. Urol Res. 2007; 35:133–137. 17396251 10.1007/s00240-007-0085-5

[22] Bhagat SK, Chacko NK, Kekre NS, Gopalakrishnan G, Antonisamy B, Devasia A. Is there a role for tamsulosin in shock wave lithotripsy for renal and ureteral calculi? J Urol. 2007; 177: 2185–2188. 17509314 10.1016/j.juro.2007.01.160

[23] Kobayashi M, Naya Y, Kino M, Awa Y, Nagata M, Suzuki H, et al Low dose tamsulosin for stone expulsion after extracorporeal shock wave lithotripsy: efficacy in Japanese male patients with ureteral stone. Int J Urol. 2008; 15:495–498. 10.1111/j.1442-2042.2008.02033.x 18422579

[24] Naja V, Agarwal MM, Mandal AK, Singh SK, Mavuduru R, Mete UK, et al Tamsulosin facilitates earlier clearance of stone fragments and reduces pain after shockwave lithotripsy for renal calculi: results from an open-label randomized study. Urology. 2008; 72: 1006–1011. 10.1016/j.urology.2008.05.035 18799202

[25] Gravas S, Tzortzis V, Karatzas A, Oeconomou A, Melekos MD. The use of tamsulozin as adjunctive treatment after ESWL in patients with distal ureteral stone: do we really need it? Results from a randomised study. Urol Res. 2007; 35: 231–235. 17609936 10.1007/s00240-007-0106-4

[26] Wang HJ, Liu K, Ji ZG, Li HZ. Application of Alpha1-adrenergic antagonist with extracorporeal shock wave lithotripsy for lower ureteral stone. Zhongguo Yi Xue Ke Xue Yuan Xue Bao. 2008; 30: 506–508. 18795629

[27] Ateş F, Eryıldırım B, Öztürk MI, Turan T, Gürbüz C, Ekinci MO, et al Does the use of doxazosin influence the success of SWL in the treatment of upper ureteral stones? A multicenter, prospective and randomized study. Urol Res. 2012; 40: 537–542. 10.1007/s00240-011-0455-x 22228043

[28] Janane A, Hamdoun A, Hajji F, Dakkak Y, Ghadouane M, Ameur A, et al Usefulness of adjunctive alpha1-adrenergic antagonists after single extracorporeal shock wave lithotripsy session in ureteral stone expulsion. Can Urol Assoc J. 2014; 8: E8–E11. 10.5489/cuaj.1261 24454608 PMC3896566

[29] Hussein MM. Does tamsulosin increase stone clearance after shockwave lithotripsy of renal stones? A prospective, randomized controlled study. Scand J Urol Nephrol. 2010; 44: 27–31. 10.3109/00365590903359916 19947900

[30] Gul U, Yaycıoglu O, Kuzgunbay B, Sarıturk C, Kayra MV, Ozkardes H. Do patients on alpha-blockers for the treatment of benign prostatic hyperplasia have better results after shockwave lithotripsy of urinary stones? J Endourol. 2013; 27: 612–616. 10.1089/end.2012.0630 23237326

[31] Wang H, Liu K, Ji Z, Li H. Effect of alpha1-adrenergic antagonists on lower ureteral stones with extracorporeal shock wave lithotripsy. Asian J Surg. 2010; 33: 37–41. 10.1016/S1015-9584(10)60007-3 20497881

[32] Cho HJ, Shin SC, Seo do Y, Min DS, Cho JM, Kang JY, et al Efficacy of alfuzosin after shock wave lithotripsy for the treatment of ureteral calculi. Korean J Urol. 2013; 54: 106–110. 10.4111/kju.2013.54.2.106 23550174 PMC3580299

[33] Park YH, Lee HE, Park JY, Lee SB, Kim HH. A prospective randomized controlled trial of the efficacy of tamsulosin after extracorporeal shock wave lithotripsy for a single proximal ureteral stone. Korean J Urol. 2013; 54: 527–530. 10.4111/kju.2013.54.8.527 23956828 PMC3742905

